# Alleviating Terminal Pediatric Cancer Pain

**DOI:** 10.3390/children8030239

**Published:** 2021-03-19

**Authors:** Karen Moody, Mohammad Baig, Veronica Carullo

**Affiliations:** 1 Department of Pediatrics, The University of Texas M.D. Anderson Cancer Center, Houston, TX 77030, USA; mbaig@mdanderson.org; 2 Department of Anesthesiology and Pediatrics, University of Mississippi Medical Center, Jackson, MS 39216, USA; vcarullo@umc.edu

**Keywords:** cancer, child, terminal pain, end-of-life

## Abstract

Terminal cancer pain remains one of the most distressing aspects of pediatric oncology practice. Opioids are the cornerstone of cancer pain management at end-of-life and fortunately, most pain at end-of-life can be managed successfully. This article presents a practical step-by-step approach to alleviating pediatric terminal cancer pain, which can be delivered across settings.

Terminal cancer pain remains one of the most distressing aspects of pediatric oncology practice. Opioids are the cornerstone of cancer pain management at end-of-life. Fortunately, most cancer pain at end-of-life (or terminal cancer pain) is responsive to opioid therapy, and providers and parents can focus on the other aspects of the dying process once a dying child’s pain is largely relieved. Total pain is the concept coined by Dame Cicely Saunders, founder of hospice model of care, to describe the physical, emotional, social, and spiritual dimensions of suffering [1]. There is no doubt that all of these dimensions impact one another and that each must be carefully assessed and addressed. Clearly, physical pain negatively impacts health-related quality of life in children with advanced cancer [2]. It is not surprising, therefore, that a “good death” as defined by dying adult patients very often includes the avoidance of pain [3]. This article will focus primarily on the physical manifestations of pain and how to alleviate them in these children.

In a small prospective study of children with advanced cancer, approximately 58% reported poor health-related quality of life on the physical subscale domain within 12 weeks of death [2]. From the adult and the adolescent and young adult (AYA) literature, risk factors for uncontrolled pain have been identified. These include: 1. cancer diagnosis (particularly solid tumors infiltrating to bone or lung), 2. hospital death (as opposed to death in home hospice or palliative care setting), 3. lack of an end-of life conversation, and 4. absence of a pain management specialist consultation [4,5]. The infiltration of tumor into bone is a common problem in pediatrics where bone and soft tissue cancer accounts for approximately 18% of cancer deaths among children 1–19 years of age [6]. Not surprisingly, these patients can present with the most challenging pain syndromes at end-of-life. The association of a hospital-based death with uncontrolled pain deserves some explanation. It is common that uncontrolled pain is what brings a dying patient to the hospital, but the pain should be relieved once the patient is admitted and the providers have access to the broad hospital armamentarium. This brings us to point 3.: “lack of an end-of-life conversation”. It is quite possible that many patients in hospitals at end-of-life have not outwardly shifted their goals to comfort care, perhaps because they have chosen not to, or perhaps because these things (goals of care) were not discussed with them in a way they could hear them. This is not a trivial point. Pain management and palliative care go hand and hand with empathic communication skills. In adults with cancer, when a poor prognosis is better understood, care tends to be more comfort-focused [7]. In children, the risk of suffering from pain at end-of-life, as reported by their bereaved parents was associated with lack of oncologist involvement and the receipt of conflicting information. On the contrary, when parents recalled having hospice conversations earlier, they reported a more peaceful death for their children [8]. Therefore, optimal pain management includes both appropriate utilization of medical interventions and investing quality time in conversations about prognosis and goals of care. 

Terminal cancer pain can present due to a variety of causes. Diagnosing the specific etiology of the pain is the first step in effective pain management. In the majority of children with terminal cancer pain, the pain is due to the cancer compressing tissues and other visceral structures. At the end-of-life, as tumor growth accelerates, the pain also rapidly accelerates. Indirect tumor effects also cause pain such as obstruction of bowel and bladder, and compression of blood vessels, such as in superior vena cava syndrome. Additional cancer-related complications also can escalate pain. For example, if bleeding occurs into an abdominal cancer, peritonitis can develop and pain will escalate, or a pathologic bone fracture can also present as an acute pain exacerbation. In addition, increased penetrability of the blood vessels in tumors coupled with increased compression of the vessels by growing tumor mass in an environment rich with inflammatory mediators can lead to extravascular pooling of fluid, such as edema, ascites, and effusions that can cause pain and dyspnea [9]. General deconditioning in advanced cancer patients which occurs due to cancer cachexia, fatigue, and inactivity can also lead to painful conditions such as myalgias, decubitus ulcers, constipation, and deep venous thromboses. Occasionally, infections such as thrush, urinary tract infections, and pneumonia (to name a few) can cause increased pain. Identifying the pain etiology is an important component to effective pain management and can also provide information related to overall disease status, e.g., cancer progression. 

In addition to the cancer, procedural interventions, such as thoracentesis, placement of central indwelling catheters, urinary catheters, nasogastric/gastric tubes, and chest tubes, can be a source of pain. Side effects of medical treatments aimed at controlling cancer can also result in painful sequelae such as myopathies, bone necrosis, neuropathies, mucositis, enterocolitis, and dermatitis. Surgical intervention can lead to incisional and neuropathic pain, infected and/or non-healing wounds, phantom limb pain and lymphedema. Radiation therapy can relieve pain but can also result in side effects such as painful burns, colitis, and mucosal ulcerations. These things can compound the underlying cancer pain if they are ineffective at controlling the cancer growth. However, if treatments help to slow the cancer they can provide some temporary relief for the underlying cancer pain. Again, discussion of goals of care need to be ongoing to appropriately balance benefits and harms of therapies with questionable benefit in the context of a terminal patient’s goals of care.

As mentioned previously, the total pain experience in a dying child includes symptoms of psychosocial and spiritual distress. For instance, anger, sadness, and guilt, can negatively influence the child’s total pain experience. Familial psychosocial distress can similarly negatively influence a child’s manifestation of pain. A complete psychosocial family evaluation can identify non-physical suffering and facilitate interventions to soothe existential and psychosocial distress.

When confronted with a child in terminal pain, a validated tool for age should be used to measure the amount of pain the child is experiencing. The gold standard for pain intensity is child self-report when possible. Most children over age 8 years can use a numerical rating scale (0–10) [10]. Children over 3 years of age do well with Faces Pain Scale-Revised [11]. When assessing infants and children 3 years of age and younger, or a child is non-verbal or developmentally delayed, a FLACC scale can be used [12]. Once the pain intensity is established, acting swiftly to alleviate pain is in order. Many children with cancer have contraindications to non-steroidal anti-inflammatory agents, and therefore opioids are commonly prescribed for pain that does not respond to acetaminophen. The majority of terminal cancer pain will necessitate the use of opioids [13]. In an inpatient acute care setting, intravenous administration of opioids is the preferred route because it works quickly, often within minutes. Determining the correct opioid dose for a patient experiencing terminal cancer pain requires a pain titration. 

A pain titration is particularly helpful to quickly bring a patient’s pain under control and to determine the optimal amount of opioid required for pain management in a patient with unyielding cancer pain in the end-of-life setting. The ideal opioid dose is defined as the minimal dose which appropriately ameliorates pain and without intolerable side effects. Although there is technically “no ceiling” dose for an opioid in absolute milligrams/kilograms, the dose that produces unwanted side effects without additional benefit is the ceiling dose for that patient at that moment. A pain titration uses intravenous (IV) doses of a short acting opioid (morphine, hydromorphone or fentanyl) given every 30 min after a concurrent pain assessment. After taking a baseline pain score (usually a score of 8–10 in this setting), the next step is to administer the lower option of a pediatric weight-based or adult-based opioid dose. Then after 15–30 min, the pain score is re-assessed. If the patient is presenting after already receiving an IV opioid, then that is considered the first dose. If the patient has received an oral opioid, then convert the dose to an IV opioid (e.g., 10 mg oral oxycodone is equivalent to 15 mg of oral morphine which is 5 mg IV morphine) and give the converted dose as is; that is, without dose reduction for incomplete cross-tolerance. The dose is not reduced because of the severity of the patient’s terminal pain. Of note, patients in renal failure should not be given morphine as the neuroexcitatory metabolites cannot be dialyzed and cross the blood brain barrier and these metabolites can cause delirium and potential blockade of mu agonism. Additional intravenous opioid doses are given depending on the subsequent pain scores in this manner: (1) if no response and/or pain is ≥7, give a higher dose by 50–100%, (2) if some response and/or pain is ≥5, administer 50–100% of the initial dose, (3) if pain score is <4, then hold dose at that time. This algorithm is continued until pain score is less than or equal to 4, or unacceptable adverse events or toxicities appear. The hourly rate of morphine can be calculated as follows: Take the total amount of opioid given to reach a pain score of ≤4 and divide that by the number of hours from the first dose of the pain titration until the time at which the patient reports a pain score of ≤4. This is the mg of morphine needed per hour to relieve this patient’s pain and can be used to calculate an equivalent daily methadone dose, a continuous morphine infusion rate, an equivalent long-acting oral opioid, or a fentanyl patch [14]. 

Methadone is often the preferred option by palliative care specialists due to its many mechanisms of action including: 1. mu, kappa and delta opioid receptor agonism, 2. N-methyl D-aspartate (NMDA) receptor antagonist, 3. weak dopaminergic activation (lower potential for euphoric effects), and 4. may prevent reuptake of serotonin and norepinephrine [15]. These properties enhance its ability to control pain compared to simple mu agonists by enabling it to relieve both nociceptive and neuropathic pain [16]. These attributes also protect patients from the development of tolerance. For these reasons, it is truly an ideal option for use at end-of-life when pain is often multifactorial and pain escalation is likely to occur. Methadone is also highly bioavailable and can be given as an aqueous solution: there are 1 mg/mL and 10 mg/mL formulations. An important feature of methadone is its long half-life, which increases with repeated doses, eventually reaching steady state after several days. Further dose adjustments should be deferred until steady state is achieved (after about 5 days). This delay in reaching steady state necessitates a strategy for breakthrough pain, which should be in place for all patients in this setting. See Table 1 for steps to convert opioids to methadone.

1. Calculate 24 h opioid use. (Can take an average of the last 3 days).
2. Convert 24 h opioid to all oral morphine (MEDD, morphine equivalent daily dose) using table above [17] (This table was adapted and modified per authors’ experience from an open access reference, and conversion for fentanyl also incorporated [17,18].)
3. Obtain baseline ECG. Consider alternative plan for QTc > 450 ms.
4. Convert MEDD to methadone using the table below [17] and reduce the result 50%. (This table was adapted and modified from an open access reference [19].)
5. Divide daily dose into 2 or 3 doses for BID or TID dosing

MEDD to Oral Methadone Conversion REDUCE by 50% for Incomplete Cross Tolerance Morphine Equivalent Daily Dose (MEDD)Mg of Oral MethadoneMg of Oral Morphine<100 mg/day14100–300 mg/day18301–600 mg/day110601–800 mg/day112801–1000 mg/day115>1000 mg/day120IV methadone is twice as potent as oral methadone

Example: Patient received 3 doses of 10 mg oxycodone orally and 6 doses of 2 mg IV morphine in previous 24 h. 3 × 10 mg oxycodone = 30 mg oxycodone × 3/2 = 45 mg oral morphine, and 6 × 2 mg = 12 mg IV morphine × 3 = 36 mg oral morphine. So 45 mg + 36 mg = 81 mg MEDD. Using the above chart, 81 mg/4 = 20.25 mg methadone. Further reduce dose by 50%. So 10 mg methadone per day or 5 mg BID.

Breakthrough pain will need to be treated with as needed medication. Additional doses of morphine at 20% of the daily morphine dose calculated and offered every 2–3 h IV prn, orally every 3–4 h, or via patient-controlled analgesia (PCA) at a morphine dose equivalent to the calculated hourly morphine rate with a lock-out of every 10 min. In children with cancer, breakthrough pain is most effectively treated with patient-controlled analgesia [20]. In the terminal setting, it is not uncommon for 80% of the daily opioid used each day to be delivered continuously via methadone or an IV opioid infusion [21]. If a patient is too young or has altered mental status precluding the use of a PCA, a PCA by proxy (pressed by parent or nurse after appropriate education) is acceptable if goals of care are comfort-focused [22]. See Table 2 for initial pediatric PCA dosing.

1. Opioid Naïve Patients:
  - Patient should be alert and demonstrate ability to administer demand dose for pain or for end-of life patients with comfort care orders, may use a by-proxy PCA. Start with above dose for PCA and set basal rate to zero.
  - Consider adding continuous (basal) dose after 12–24 h if using frequent demand doses or if pain not adequately controlled. Suggested basal dose is 50–80% of average hourly dose for end-of-life patients.
2. Opioid tolerant patients (currently receiving opioid therapy):
  - Calculate total dose of opioid (scheduled and breakthrough doses) used in the previous 24 h period to establish the new continuous rate and follow example as above.

Clinician boluses may be given to patients by the bedside nurse at a dose of 2× the PCA dose. These are indicated if pain is uncontrolled which may occur after a long nap, or long period of distraction. If tumor is growing or bleeding causing re-emergence of uncontrolled pain, the patient may need an upward adjustment of the PCA immediately following the clinician bolus.

Re-assessment of pain should occur at frequent intervals daily and opioid doses should be titrated up for escalations in pain and in amounts proportional to the previous 24-h opioid use. It is not unusual for a dying child to require frequent increases in opioid doses, sometimes even twice daily when nearing death [23]. The basal opioid infusion can be increased as frequently as every 12–24 h to maintain the PCA dose less than 2 per hour while awake, and provide ample analgesia to enable the child to sleep peacefully. A bolus dose administered by a clinician of approximately two times the PCA dose can be provided every 1–2 h while awaiting therapeutic opioid levels [14].

When pain remains uncontrolled after appropriate escalation or rotation of opioids, and/or there are continuing dose-limiting side effects without relief of pain, then the patient may be suffering from opioid-refractory or intractable cancer pain. These patients are often experiencing severe neuropathic pain, opioid tolerance and/or opioid-induced hyperalgesia [24]. If spinal cord compression is suspected or evident, radiation and/or steroids should be considered as an initial intervention. Relatively low doses of methadone and/or ketamine, due to their *N*-methyl-d-aspartate (NMDA) receptor antagonism can effectively rescue patients from intractable pain states [25]. Ketamine can sometimes be administered at home via infusion or orally. Lidocaine is a third option that can be administered intravenously to address acute neuropathic pain refractory to opioids in the hospital setting in patients with short life expectancy [26].

Epidural and peripheral nerve blocks are a potential next step when all other interventions have failed to provide adequate relief [27]. These interventions are generally performed by pain specialists trained in anesthesia and pain management. Common contraindications to this type of interventional pain management, such as low blood counts, are relative in the end-of-life setting. However, as with any intervention with considerable risk in the end-of-life setting, a clear and compassionate conversation in which the benefits and burdens are considered within the context of life expectancy and goals of care is necessary. An important component to ongoing goals of care conversations with parents of children with cancer and, potentially the children themselves, is location of death. In order to create an advance care plan and align care with patient’s goals, including place of death at end-of-life, it is important to have this information [28]. Fortunately, most pain management plans can be translated to the home setting with the assistance of home hospice programs in the United States, or to free- standing pediatric hospices in countries such as England, where those exist. The use of continuous ambulatory delivery devices (CADDs) allow for PCA use outside of hospitals [29]. Neuraxial blockade catheters and peripheral nerve block catheters can also be kept in place outside of a hospital setting [27]. Subcutaneous continuous opioid infusions can be used when patients do not have a central venous catheter access [30]. A partnership between palliative care specialists and pain management specialists can facilitate alleviation of pain and death in the desired location [31].

Once the opioid backbone and breakthrough dose of the treatment plan is established, a consideration for the use of pain adjuvants is necessary. Adjuvants can be opioid-sparing and allow for better overall pain relief by addressing other specific causes of pain such as peripheral neuropathy, muscle spasms, focal bone lesions, visceral spasms of bladder or bowel, etc. A full review of adjuvants is beyond the scope of this review. However, it suffices to say that one should become familiar with at least 1–2 medications in each class including gabapentinoids, serotonin/norepinephrine reuptake inhibitors, and tricyclic antidepressants for neuropathic pain; muscle relaxants, such as cyclobenzaprine, methocarbamol or diazepam; visceral antispasmodics such as hyoscyamine and dicyclomine; and topical analgesics such as lidocaine patches, lidocaine jelly, and compounded creams containing mixtures of local anesthetics, neuropathic agents, muscle relaxants and/or non-steroidal agents. Other adjuvant interventions may include dexamethasone, anxiolytics, clonidine, bisphosphonates, and short course radiation [32]. Non-pharmacological adjuvants such as ice, heat, mind-body interventions and distraction should also be used as appropriate [33]. Effectively addressing other distressing symptoms such as insomnia and anxiety also contribute to better pain relief [4,14].

Opioid side effects are often an unavoidable consequence of their use; therefore, anticipation of side effects with “as needed” orders may be indicated. Common symptoms include constipation, sedation, pruritis, nausea and urinary retention. Opioid rotation can alleviate the vast majority of side effects as well without compromising pain control [34]. In some cases, the “as needed” orders may need to be converted to around the clock, especially for constipation, when symptoms are persistent. It is extremely important to monitor and treat constipation in these patients as well as to have a low index of suspicion for overflow encopresis/stool impaction.

In summary, terminal cancer pain is a frequent burden of children dying of cancer. Every effort should be made to regularly assess and to alleviate this pain in the context of a patient’s values, prognosis, and goals of care. There are many ways to address pain effectively and most interventions can be translated to the location of choice, including at home with hospice care or in a hospice facility.

## Figures and Tables

**Table 1:** Initiation of methadone.

IV Morphine to PO Morphine = 1:3
IV Hydromorphone to PO Hydromorphone = 1:5
PO Hydromorphone to PO Morphine = 1:4
IV Hydromorphone to IV Morphine = 1:5
PO Oxycodone to PO Morphine = 2:3
IV Fentanyl to IV Morphine = 0.1–0.2 mg:10 mg

**Table 2:** Initial PCA (patient-controlled analgesia) dosing.

Opioid	Demand Dose (PCA)	Lock-Out Time (Minutes)	Clinician Bolus Dose	Basal Rate (Continuous Infusion)
Morphine (mg)	0.025 mg/kg (max 2 mg)	10–30 min	0.05–0.1 mg/kg (max 2 mg)	0
Hydromorphone (mg)	0.005 mg/kg (max 0.3 mg)	10–30 min	0.01–0.02 mg/kg (max 1 mg)	0
Fentanyl (mcg) *	0.25 mcg/kg/dose (max 25 mcg)	10–30 min	0.5–1 mcg/kg/dose (max 50 mcg)	0

* Example: The 12 h total morphine demand dose is 24 mg, calculate continuous dose as 20/12 = 2 mg/h then 2 × 0.5 (50%) = 1 mg/h basal rate. Then adjust the PCA dose to 1× the basal rate, in this case, 1 mg every 10 min.

## Data Availability

Data are contained within the article.
